# *Fusarium guttiforme* Inoculation Methods for Resistance Phenotyping in a Segregating Pineapple Population

**DOI:** 10.3390/mps9050141

**Published:** 2026-09-26

**Authors:** Marcelo Serafim de Andrade Junior, Marcelo Vivas, Adrielim Santiago Lima, Luciana Pereira Pinto, Davi Cardinot de Castro Pereira, Ana Lucia Rangel de Souza, Gizeli Santiago Lima, Roberto Ramos-Sobrinho, Rafael Nunes de Almeida, Tiago Silva Jorge, Marta Simone Mendonça Freitas, Almy Junior Cordeiro de Carvalho

**Affiliations:** 1 Laboratory of Agricultural Engineering, Center for Agricultural Sciences and Technologies, Universidade Estadual do Norte Fluminense Darcy Ribeiro—UENF, Campos dos Goytacazes 28013-602, RJ, Brazil; marcelojunior.a@hotmail.com (M.S.d.A.J.); adrielimsantiago@gmail.com (A.S.L.); lucianapereirap7@gmail.com (L.P.P.); davicp330@gmail.com (D.C.d.C.P.); rangelana.agro@gmail.com (A.L.R.d.S.); gizelisantiago4321@gmail.com (G.S.L.); tiago.uenf@hotmail.com (T.S.J.); 2 Laboratory of Entomology and Plant Pathology, Center for Agricultural Sciences and Technologies, Universidade Estadual do Norte Fluminense Darcy Ribeiro—UENF, Campos dos Goytacazes 28013-602, RJ, Brazil; ramosrs@uenf.br; 3 Capixaba Institute of Research, Technical Assistance and Rural Extension—Incaper, Vitória 29052-010, ES, Brazil; rafael.almeida@incaper.es.gov.br; 4 Laboratory of Crop Science, Center for Agricultural Sciences and Technologies, Universidade Estadual do Norte Fluminense Darcy Ribeiro—UENF, Campos dos Goytacazes 28013-602, RJ, Brazil; msimone@uenf.br (M.S.M.F.); almy@uenf.br (A.J.C.d.C.)

**Keywords:** plant breeding, fusariosis, second-generation hybrid

## Abstract

Pineapple (*Ananas comosus* (L.) Merr.) is an economically important crop in Brazil, but its production is constrained by fusariosis caused by *Fusarium guttiforme*. This study evaluated two inoculation and resistance-phenotyping procedures in a segregating population derived from a cross between the susceptible cultivar ‘Pérola’ and the resistant cultivar ‘BRS Imperial’. The experiment was conducted using an augmented block design with 73 hybrids and the two parents as common checks. Plants were evaluated using detached-leaf and direct stem inoculation procedures, and lesion area was quantified using ImageJ. Significant genotypic effects were detected for all evaluated traits (*p* < 0.01). Stem lesion area showed a higher coefficient of determination of genotypic effects (h^2^g = 0.67) than the leaf traits (h^2^g = 0.31), indicating greater discrimination among segregating individuals under the evaluated conditions. Maximum leaf lesion area and leaf AUDPC were strongly correlated (ρ = 0.954), whereas neither leaf trait was significantly associated with stem lesion area. IBP2_70, IBP2_24, and IBP1_3 showed adjusted genotypic values lower than that of the resistant cultivar ‘BRS Imperial’ and were identified as promising candidates for subsequent validation. These results show that both procedures detect genotypic variation but provide non-equivalent phenotypic information.

## 1. Introduction

Pineapple (*Ananas comosus* (L.) Merr.) is a tropical fruit crop of considerable economic importance worldwide, including in Brazil, where production is intended predominantly for the fresh-fruit market. The cultivar ‘Pérola’ occupies a prominent position in Brazilian pineapple production because of its broad adaptation and desirable sensory attributes, including juicy flesh, sweet taste, intense aroma, and low acidity [1,2]. However, plant development, yield, and fruit quality can be affected by several biotic and abiotic factors, among which diseases are particularly important.

Fusariosis, also known as gummosis or resinous disease, is one of the major pineapple diseases in Brazil and is caused by *Fusarium guttiforme* Nirenberg & O’Donnell [3]. The disease occurs at different developmental stages and affects planting material, adult plants, and fruits. In young plants, symptoms include stem lesions, infection of the basal nonchlorophyllous region, foliar alterations, tissue decay, and gum exudation. In fruits, peel discoloration, tissue rot, and intense gum exudation may occur, rendering them unsuitable for marketing [4]. In susceptible cultivars, management relies on cultural practices, healthy planting material, and repeated fungicide applications, which may increase production costs and provide variable control efficacy [4,5]. Therefore, developing resistant cultivars is an important and sustainable strategy for reducing losses and dependence on chemical control.

The cultivar ‘BRS Imperial’ was developed as an alternative resistant to *F. guttiforme* and combines desirable agronomic and fruit-quality traits [5], although it has not achieved the same consumer acceptance as ‘Pérola’. Inoculation assays confirmed its resistant response across different pathogen isolates and substrate conditions, whereas ‘Pérola’ was highly susceptible [4,6]. Segregating populations derived from crosses between these parents are promising materials for studying and selecting resistance because they combine contrasting disease responses and complementary agronomic traits. In a population derived from this cross, Lira Júnior et al. [4] identified resistant, moderately resistant, and susceptible individuals, demonstrating useful variability for selection. Recent breeding studies have also addressed the joint selection of spineless plants and fusariosis resistance [7], including the development of ‘Unemat Rubi’ from ‘Imperial’ × ‘Pérola’ [8]. Field evaluation of resistant hybrids in southern Bahia further showed the importance of assessing yield and fruit quality across cultivation sites [9]. These studies place resistance phenotyping within a broader selection process that also considers agronomic performance.

Successful breeding for disease resistance depends not only on generating genetic variability but also on the accurate, consistent, and reproducible phenotyping of segregating individuals. Inoculation procedures should produce adequate disease levels and reliably discriminate genotypic responses. Factors such as the tissue evaluated, inoculation method and site, inoculum concentration, and experimental conditions may influence symptom intensity and genotype classification [10,11,12]. In the pineapple–*F. guttiforme* pathosystem, procedures based on seedling and detached-leaf inoculation have been described. In pineapple, the D leaf corresponds to the youngest fully developed leaf of the rosette and is commonly used as a standardized reference tissue. Garcia et al. [10] identified detached D leaves as a suitable tissue for rapid and economical assessment of fusariosis symptoms. Based on this previous study, the D leaf was selected here as a standardized tissue for detached-leaf inoculation, allowing the same developmental leaf type to be evaluated across the segregating individuals. Lira Júnior et al. [4] used seedling immersion inoculation. The present study was not designed to compare all inoculation procedures available for pineapple fusariosis. Instead, we focused on two contrasting approaches that could provide complementary phenotypic information within the same segregating population: a rapid detached-leaf assay and direct stem inoculation of intact plants. Seedling or slip immersion methods have previously been used to evaluate pineapple responses to *F. guttiforme*, but these approaches were outside the specific scope of the present comparison. Despite these advances, information remains limited regarding the ability of assessments performed on different tissues to detect and quantify genotypic variation within the same segregating population. Accordingly, further comparative validation is needed to define the scope and limitations of each procedure for quantitative phenotyping in segregating pineapple populations. This methodological concern also arises in wheat: Syed et al. [13] compared spray, point, and grain-spawn inoculation and showed that the procedures assess different aspects of resistance to *Fusarium* head blight. Such findings support evaluating assay performance rather than assuming equivalence between inoculation methods.

The pineapple breeding program of Universidade Estadual do Norte Fluminense Darcy Ribeiro (UENF) seeks to develop fusariosis-resistant genotypes that retain the desirable agronomic and fruit-quality attributes of ‘Pérola’. Accordingly, this study aimed to evaluate two *F. guttiforme* inoculation and phenotyping procedures—detached D-leaf inoculation and direct stem inoculation—for their ability to detect genotypic variation in a segregating population derived from the cross between ‘Pérola’ and ‘BRS Imperial’. We also evaluated agreement among the traits obtained using the two procedures and identified candidate genotypes for subsequent selection and validation of fusariosis resistance.

## 2. Materials and Methods

### 2.1. Plant Material and Experimental Design

The plant material comprised 73 segregating pineapple individuals (*Ananas comosus* (L.) Merr.) obtained from seeds resulting from crosses between parents belonging to the ‘Pérola’ cultivar and one clone of ‘BRS Imperial’. The ‘Pérola’-type materials used as parents belonged to a set of 39 accessions collected from small farms in the Northern Rio de Janeiro mesoregion, encompassing municipalities in northern Rio de Janeiro State and southern Espírito Santo State, Brazil. The ‘BRS Imperial’ clone, maintained at the Laboratory of Agricultural Engineering of Universidade Estadual do Norte Fluminense Darcy Ribeiro (UENF), was used as the resistant parent. This cultivar was developed by the Embrapa Cassava and Fruits breeding program from the cross ‘Perolera’ × ‘Smooth Cayenne’ and is resistant to fusariosis caused by *Fusarium guttiforme* [5,6]. In addition to the 73 segregating individuals, the parental cultivars ‘Pérola’ and ‘BRS Imperial’ were included as common checks, totaling 75 evaluated genotypes.

The segregating individuals and parental cultivars were grown individually in 5 L pots containing a 1:1 (*v*/*v*) mixture of sand and manure. After homogenization, the substrate was placed in autoclavable plastic bags and heat-treated in an autoclave at 120 °C for 60 min. Following treatment, the substrate remained in the sealed bags for seven days before being distributed among the pots.

The experiment was conducted using an augmented block design comprising three blocks. The 73 segregating individuals were evaluated without replication, with one plant per plot, whereas ‘Pérola’ and ‘BRS Imperial’ were used as common checks and included in every block.

Plants were maintained in a plastic-covered greenhouse at the UENF Research Support Unit in Campos dos Goytacazes, Rio de Janeiro State, Brazil. Plants were fertilized monthly by alternating 5 g of urea per plant with 5 g per plant of NPK 20–0–20 fertilizer. Consequently, each fertilizer source was applied at 60-day intervals. Plants were irrigated manually as needed.

### 2.2. Fusarium guttiforme Isolate

Both inoculation procedures used *F. guttiforme* isolate CF/UENF512. The isolate was obtained by indirect isolation from a symptomatic pineapple fruit collected in a commercial production area in the municipality of São Francisco de Itabapoana, Rio de Janeiro State, Brazil.

The isolate was identified based on morphological characteristics and phylogenetic analysis of partial translation elongation factor 1-α and β-tubulin gene sequences deposited in GenBank under accession numbers OR722688 and OR722708, respectively [14]. Its pathogenicity had previously been confirmed by inoculating leaves and fruits of the susceptible cultivar ‘Pérola’ [14].

To obtain cultures for the assays, the isolate was reactivated and multiplied on potato dextrose agar (PDA) and incubated at 25 ± 2 °C under a 12 h photoperiod for 14 days [14]. Although the same isolate was used in both procedures, inoculum preparation differed between them. Mycelium collected from the margins of actively growing colonies was used for detached D-leaf inoculation, whereas a conidial suspension prepared from colonies of the same isolate grown on PDA was used for direct stem inoculation.

### 2.3. Detached D-Leaf Inoculation Method

#### 2.3.1. Leaf Selection and Preparation

The length of the D leaf of each plant was measured monthly after transplanting. When the D leaf reached approximately 40 cm, it was manually detached from the plant at the basal region and collected for the fusariosis-resistance assay (Figure 1). After collection, leaves were surface-disinfested in 1% sodium hypochlorite solution for 2 min and then rinsed twice with sterile distilled water. Leaves were standardized by retaining an approximately 20 cm segment measured from the leaf base and containing the transition between nonchlorophyllous and chlorophyllous tissues.

The segments were placed individually in previously sanitized plastic containers prepared to maintain humidity and reduce desiccation during the assessment period.

#### 2.3.2. Leaf Inoculation

The detached-leaf inoculation procedure was based on the approach described by Garcia et al. [10], with adaptations to the plant material and assessment conditions used in the present study. Inoculation was performed at the transition between the nonchlorophyllous and chlorophyllous portions of the D leaf (Figure 1). To prepare the inoculum, a previously sterilized wooden toothpick was placed in contact with the margin of a 14-day-old colony of *F. guttiforme* CF/UENF512 grown on PDA, allowing mycelium from the actively growing region to adhere to the toothpick.

The mycelium-bearing toothpick was used to make a single superficial puncture in the leaf tissue at the previously defined region. Punctures were standardized and did not pass completely through the leaf or reach the opposite surface. After inoculation, leaf segments were returned to their respective plastic containers, where they remained throughout the assessment period.

As negative controls, leaf segments of ‘Pérola’ and ‘BRS Imperial’ were subjected to the same puncture procedure using sterile wooden toothpicks without pathogen mycelium.

#### 2.3.3. Disease Severity Assessment

Lesion development was assessed at 7, 14, 21, and 30 days after inoculation. At each assessment, leaf segments were removed from the containers and photographed individually against a uniform blue background. Leaf orientation, camera-to-sample distance, and lighting conditions were kept as constant as possible. Images were analyzed using ImageJ software (versão 1.54k, National Institutes of Health, Bethesda, MD, USA) to determine lesion area.

Disease progression in leaves was characterized by the area under the disease progress curve (AUDPC), calculated by trapezoidal integration based on the four assessments:

$$AUDPC=\sum_{i=1}^{n-1}\left(\frac{y_{i}+y_{i+1}}{2}\right)\left(t_{i+1}-t_{i}\right),$$

where *y_i_* and *y_i+1_* are the lesion areas observed at two consecutive assessments; *t_i_* and *t_i+1_* are the corresponding assessment times, in days; and n is the total number of assessments.

In addition to AUDPC, maximum lesion area was determined as the largest lesion-area value recorded for each genotype across the four assessments.

### 2.4. Direct Stem Inoculation Method

The second procedure consisted of directly inoculating a conidial suspension of *F. guttiforme* CF/UENF512 into the basal stem region of intact pineapple plants (Figure 2). As no standardized published protocol for direct stem inoculation was available, the procedure was developed from preliminary assays conducted in our laboratory. The present study standardized key methodological steps, including inoculum preparation and concentration, inoculum volume, wound depth, inoculation site, and lesion assessment, to provide a reproducible protocol for future studies.

#### 2.4.1. Preparation of the Conidial Suspension

For stem inoculation, isolate CF/UENF512 was grown on PDA at 25 °C under a 12 h photoperiod for 14 days. Ten milliliters of sterile distilled water was added to the surface of each colony, which was gently scraped using a Drigalski spatula. The resulting suspension was filtered through two layers of cheesecloth. Conidial concentration was determined using a Neubauer chamber and adjusted to 1 × 10^4^ conidia mL^−1^.

#### 2.4.2. Plant Wounding and Inoculation

Inoculation was performed approximately 11 months after establishment of the experiment. Leaves surrounding the inoculation site were initially moved aside to provide access to the basal stem region. A single puncture approximately 5 mm deep was made in each plant using a previously sterilized metal pin fitted with a wooden limiter, thereby standardizing wound depth (Figure 2).

Immediately after puncturing, 5 mL of a suspension containing 1 × 10^4^ conidia mL^−1^ was applied directly to the opening using a sterile syringe. As negative controls, plants of ‘Pérola’ and ‘BRS Imperial’ were subjected to the same puncture procedure and received 5 mL of sterile distilled water without pathogen conidia.

#### 2.4.3. Assessment of Internal Lesions

Plant responses were assessed destructively at 120 days after inoculation. Plants were removed from the pots, and leaves and roots were removed to expose the stem. Each stem was then cut longitudinally through the inoculation point to expose the internal extent of the lesion.

The two stem halves were positioned side by side, with the cut surfaces facing upward, against a uniform dark background. A graduated ruler was placed in the same plane as the evaluated surfaces and used as a reference for spatial calibration of the images (Figure 2). Photographs were obtained while keeping sample orientation, camera distance, and lighting conditions as constant as possible.

Images were analyzed using ImageJ to determine the lesion area on each longitudinal surface. The value assigned to each plant was the mean area measured on the two stem halves.

### 2.5. Statistical Analysis and Selection of Promising Individuals

Analyses were performed separately for the three variables used to characterize the inoculation response: maximum lesion area on detached D leaves, leaf AUDPC, and stem lesion area. Considering the augmented block design, data were analyzed using linear mixed models and the REML/BLUP procedure implemented in Selegen-REML/BLUP, model 76, which is appropriate for augmented block experiments conducted at one location with one plant per plot and common checks treated as fixed effects [15]. The following model was fitted for each variable:

y=Xf+Zg+Wb+e,

where *y* is the vector of observations; f is the vector of fixed effects, including the overall mean and the effects of the common checks ‘Pérola’ and ‘BRS Imperial’; *g* is the vector of random genotypic effects of the segregating individuals; *b* is the vector of random block effects; and *e* is the vector of random errors. *X*, *Z*, and *W* are the corresponding incidence matrices.

Variance components were estimated by restricted maximum likelihood (REML), and genotypic effects were predicted by best linear unbiased prediction (BLUP). The significance of genotypic and block effects was evaluated using the likelihood-ratio test (LRT), calculated as the difference between the deviances of the full model and the respective reduced model, from which the effect under evaluation was excluded. LRT values were compared with the chi-square distribution with one degree of freedom, using a 1% significance level (*p* < 0.01).

For each variable, the genotypic variance (Vg), variance associated with block effects (Vblock), and residual variance (Ve) were estimated. Total phenotypic variance (Vf) was obtained as follows:

$$V_{f}=V_{g}+V_{block}+V_{e}.$$

The coefficient of determination of genotypic effects (h^2^g), coefficient of determination of block effects (c^2^block), and proportion of residual variance (c^2^res) were also calculated as follows:

$$h_{g}^{2}=\frac{V_{g}}{V_{f}};\;\;c_{block}^{2}=\frac{V_{block}}{V_{f}};\;\;c_{res}^{2}=\frac{V_{e}}{V_{f}}.$$

Agreement among the responses obtained using the two inoculation procedures was examined using Spearman’s rank correlation. Associations among maximum leaf lesion area, leaf AUDPC, and stem lesion area were evaluated. The primary analysis included only the 73 segregating individuals. As a sensitivity analysis, coefficients were also estimated after including the mean values of the parental cultivars ‘Pérola’ and ‘BRS Imperial’, totaling 75 genotypes. The *p* values for the three correlations were adjusted using the Benjamini–Hochberg procedure to control the false discovery rate [16].

Individuals were ranked using their predicted genotypic effects (g) and corresponding adjusted genotypic values, calculated as follows:

$$\hat{\mu +g}=\hat{\mu }+\hat{g},$$

where ***μ*** is the adjusted overall mean and ***g*** is the predicted genotypic effect of each individual. Because smaller lesion areas represent more favorable responses to inoculation, candidate identification was based on adjusted genotypic values for stem lesion area. Segregating individuals with adjusted genotypic values equal to or lower than that of the resistant cultivar ‘BRS Imperial’ were considered promising. This criterion was used for preliminary selection of candidates for subsequent validation and does not constitute a definitive classification of genotype resistance.

## 3. Results

### 3.1. Ability of the Procedures to Detect Genotypic Variation

Deviance analysis revealed significant genotypic effects for all three evaluated traits (Table 1). Likelihood-ratio test (LRT) values were 9.86 for maximum leaf lesion area, 8.45 for leaf AUDPC, and 7.30 for stem lesion area, all significant at the 1% probability level. These results demonstrate that both inoculation procedures detected genotypic variation among the segregating individuals. In contrast, block effects were not significant (LRT ranging from 0.07 to 1.17), and their relative contribution to phenotypic variance ranged from 1% to 5%.

Variance components estimated by REML differed among traits (Table 2). For leaf traits, genotypic variance was 2.23 for maximum lesion area and 247.20 for AUDPC, accounting for 31% of total phenotypic variance in both cases (h^2^g = 0.31). The residual proportion was 0.65 for maximum lesion area and 0.68 for AUDPC. Stem lesion area had a genotypic variance of 0.69, residual variance of 0.29, coefficient of determination of genotypic effects of 0.67, and residual proportion of 0.28. Thus, under the conditions of this experiment, stem lesion assessment showed a larger relative contribution of genotypic effects and a smaller proportion of residual variance than the leaf traits.

### 3.2. Consistency and Agreement Between Resistance-Phenotyping Procedures

Maximum lesion area and leaf AUDPC were strongly and positively correlated (ρ = 0.954; pFDR < 0.0001), indicating a high level of consistency and very similar genotype rankings within the detached-leaf procedure. The sensitivity analysis including parental means yielded a similar correlation (ρ = 0.955), confirming the robustness of this association (Figure 3).

In contrast, stem lesion area was not significantly associated with maximum leaf lesion area (ρ = 0.080; pFDR = 0.693) or leaf AUDPC (ρ = 0.047; pFDR = 0.693). Thus, although both inoculation procedures detected significant genotypic variation, they did not provide consistent genotype rankings across tissues. Moreover, stem lesion area showed a higher coefficient of determination of genotypic effects (h^2^g = 0.67) and a lower residual proportion (c^2^res = 0.28) than the leaf traits (h^2^g = 0.31; c^2^res = 0.65–0.68). Therefore, under the conditions of this experiment, the stem inoculation procedure showed greater ability to discriminate genetic variation among the segregating individuals. Taken together, these results indicate that detached-leaf and stem inoculation are not interchangeable procedures for resistance identification and may capture distinct components of the pineapple response to *Fusarium guttiforme*.

### 3.3. Prediction of Genotypic Values and Candidate Identification

Predicted genotypic effects for stem lesion area varied widely among the segregating individuals (Table 3). Of the 73 evaluated individuals, 40 (54.8%) had negative genotypic effects, indicating a predicted lesion area below the overall mean, although this result alone does not provide evidence of resistance.

Comparison with the parents showed that 30 individuals (41.1%) had adjusted genotypic values lower than that of ‘Pérola’ (μ + g = 1.9590). Of these, 27 were positioned between ‘Pérola’ and ‘BRS Imperial’ (μ + g = 1.1031). Three individuals—IBP2_70 (μ + g = 0.9181), IBP2_24 (μ + g = 1.0661), and IBP1_3 (μ + g = 1.0768)—had values lower than that of the resistant cultivar and were identified as promising candidates for subsequent validation (Figure 4).

## 4. Discussion

The significant genotypic effects observed for all three traits demonstrate that both inoculation procedures detected variation in the segregating population. Contrasting responses were also observed by Lira Júnior et al. [4] among F_1_ progeny from the cross ‘BRS Imperial’ × ‘Pérola’, in which resistant, moderately resistant, and susceptible genotypes were identified after inoculation with *F. guttiforme*. In that study, ‘Pérola’ developed severe symptoms and seedling death, whereas ‘BRS Imperial’ developed neither foliar symptoms nor internal stem lesions, confirming the contrast between the parents used in the present study. However, Lira Júnior et al. [4] used slips, immersion inoculation, and a categorical symptom scale, whereas the present study quantitatively measured lesion area. These differences preclude direct comparisons between the proportions of individuals assigned to each reaction class.

Variance components revealed differences in the discriminatory ability of the traits. Stem lesion area had a coefficient of determination of genotypic effects of 0.67, higher than the value of 0.31 observed for the leaf traits, as well as a lower residual proportion. These results indicate that, under the evaluated conditions, stem inoculation had a greater ability to discriminate genotypic variation. This conclusion should be distinguished from the operational efficiency of the protocol. When comparing leaf types and inoculation methods, Garcia et al. [10] considered detached D leaves the fastest, most economical, and most satisfactory procedure for reproducing symptoms and recommended assessments between 10 and 20 days after inoculation. Thus, the results of the two studies are not necessarily conflicting: detached leaves may offer advantages in time, cost, and sample throughput, whereas the stem procedure showed a larger relative contribution of genotypic effects in the present experiment.

A similar distinction between rapid screening and symptom reproduction was reported for *Coffea canephora*. Da Rocha et al. [17] found that conidial inoculation of leaf disks was time-saving, whereas mycelial inoculation of wounded stems was the most effective approach for inducing stem canker symptoms. Although the host and *Fusarium* species differ from those studied here, this comparison illustrates why the practical value of an assay depends on its intended endpoint.

The strong correlation between maximum lesion area and AUDPC shows that the two leaf traits provided largely redundant information. Garcia et al. [10] also used lesion measurements and AUDPC to characterize the responses of pineapple accessions on detached D leaves and identified 10–20 days after inoculation as an appropriate period for discriminating among materials. Together, these results indicate potential for reducing the number of assessments in the leaf protocol. However, maximum lesion area in the present study was defined from four successive assessments. Therefore, a single assessment time cannot yet be recommended without first determining which time point preserves genotype ranking and agreement with AUDPC.

The comparison of genotype rankings across procedures showed high consistency within the detached-leaf assessment but low consistency between detached-leaf and stem inoculation. The lack of association between leaf traits and stem lesion area indicates that the two procedures should not be considered interchangeable for resistance identification. In addition, stem lesion area showed a higher coefficient of determination of genotypic effects (h^2^g = 0.67) and a lower residual proportion (c^2^res = 0.28) than the leaf traits (h^2^g = 0.31; c^2^res = 0.65–0.68). Therefore, under the conditions of this experiment, stem inoculation provided greater discrimination of genetic variation among the segregating individuals. This difference should be interpreted as greater discriminatory ability within the evaluated experimental conditions rather than as evidence that stem inoculation is universally superior. The divergence between procedures may be related to the specificity of the tissue evaluated and to differences in inoculum application and quantity. Garcia et al. [10] showed that leaf type and inoculation procedure affect symptom expression. Furthermore, Almeida et al. [18] detected an interaction between pineapple genotypes and *F. guttiforme* isolates for root-system traits, showing that infection responses may vary according to the combination of host, isolate, and organ evaluated. Therefore, the lack of correlation observed here may reflect distinct components of the host response rather than failure of either procedure.

Recent studies provide contrasting evidence on the predictive value of detached tissues. In wheat, Rafiei et al. [19] found that seedling and coleoptile assays agreed with head-infection assessments, whereas detached leaves inconsistently distinguished resistant and susceptible genotypes. Conversely, Kirkby et al. [20] developed a detached-leaf assay for industrial hemp that differentiated susceptibility to *Botrytis cinerea* and produced responses comparable to whole-plant tests. These contrasting outcomes emphasize that correspondence between detached-tissue and whole-plant assays must be established within each pathosystem. They do not establish the biological cause of the low leaf–stem association observed in the present experiment.

Accordingly, detached-leaf assessment should be interpreted as a rapid procedure for characterizing the foliar response, but not as a substitute for or predictor of the stem response. Although stem inoculation was more time-consuming, destructive, and dependent on greater space for plant growth, it showed greater discriminatory ability under the evaluated conditions. For application in breeding programs, the procedures serve different purposes: detached leaves enable rapid assessment of the local tissue reaction, whereas the stem provides a measure related to internal plant colonization.

Previous studies suggest that resistance to pineapple fusariosis may have relatively simple inheritance, controlled by one or a few genes, with resistance dominant over susceptibility, as discussed by Lira Júnior et al. [4]. This model may help explain the presence of contrasting individuals in populations derived from resistant and susceptible parents. However, the continuous distribution of genotypic values for lesion area observed in the present study indicates that phenotypic disease expression is also influenced by the inoculation procedure and residual components. Therefore, these results should not be interpreted as evidence of the inheritance model because the experiment was not designed to test Mendelian segregation.

Adjusted genotypic values identified IBP2_70, IBP2_24, and IBP1_3 as candidates with less lesion development than the resistant reference ‘BRS Imperial’. The identification of individuals with contrasting responses is consistent with Lira Júnior et al. [4], who found six resistant, five moderately resistant, and seven susceptible genotypes among 18 selected hybrids from the same parental cross. However, differences in protocols, isolates, and classification criteria prevent direct comparison of the frequencies observed in the two studies. The development of ‘Unemat Rubi’ [8] also illustrates that identifying fusariosis-resistant material is only one step toward cultivar release. The present candidates therefore require subsequent evaluation of fruit quality and agronomic performance, in addition to confirmation of their disease response.

The images in Figure 4 illustrate the internal lesions observed in the three candidates and parental cultivars, but classification was based on adjusted genotypic values rather than visual inspection alone. Although promising, these findings represent a preliminary stage because each individual was represented by a single plant and only isolate CF/UENF512 was used. This limitation is relevant because Ribeiro et al. [14] demonstrated variation in aggressiveness among *F. guttiforme* isolates from northern Rio de Janeiro State, whereas Almeida et al. [18] observed responses dependent on the cultivar–isolate combination. Candidate resistance should therefore be confirmed using clonally propagated plants, a larger number of replicates, and genetically and phenotypically distinct isolates. The importance of broader pathogen sampling is also illustrated in soybean, where Wu et al. [21] documented differences in aggressiveness among *Fusarium* isolates and evaluated cultivar responses to multiple species. For pineapple, such validation should focus on representative *F. guttiforme* isolates rather than extrapolating resistance from the single isolate used here.

## 5. Conclusions

Both inoculation procedures detected genotypic variation in the response of segregating pineapple individuals to *Fusarium guttiforme*. Maximum lesion area and leaf AUDPC showed high agreement, indicating that these traits provide largely related information. However, the leaf traits were not associated with stem lesion area and should therefore not be used as predictors or substitutes for the internal plant response.

Under the conditions of this experiment, both phenotyping procedures were effective in detecting genotypic variation in response to *Fusarium guttiforme*. Stem lesion area provided greater discrimination among segregating individuals than the detached-leaf traits. IBP2_70, IBP2_24, and IBP1_3 were identified as promising candidates for subsequent selection stages. Definitive classification of these candidates as resistant requires further validation using clonally propagated plants, additional replication, and different *F. guttiforme* isolates.

## Figures and Tables

**Figure 1:**
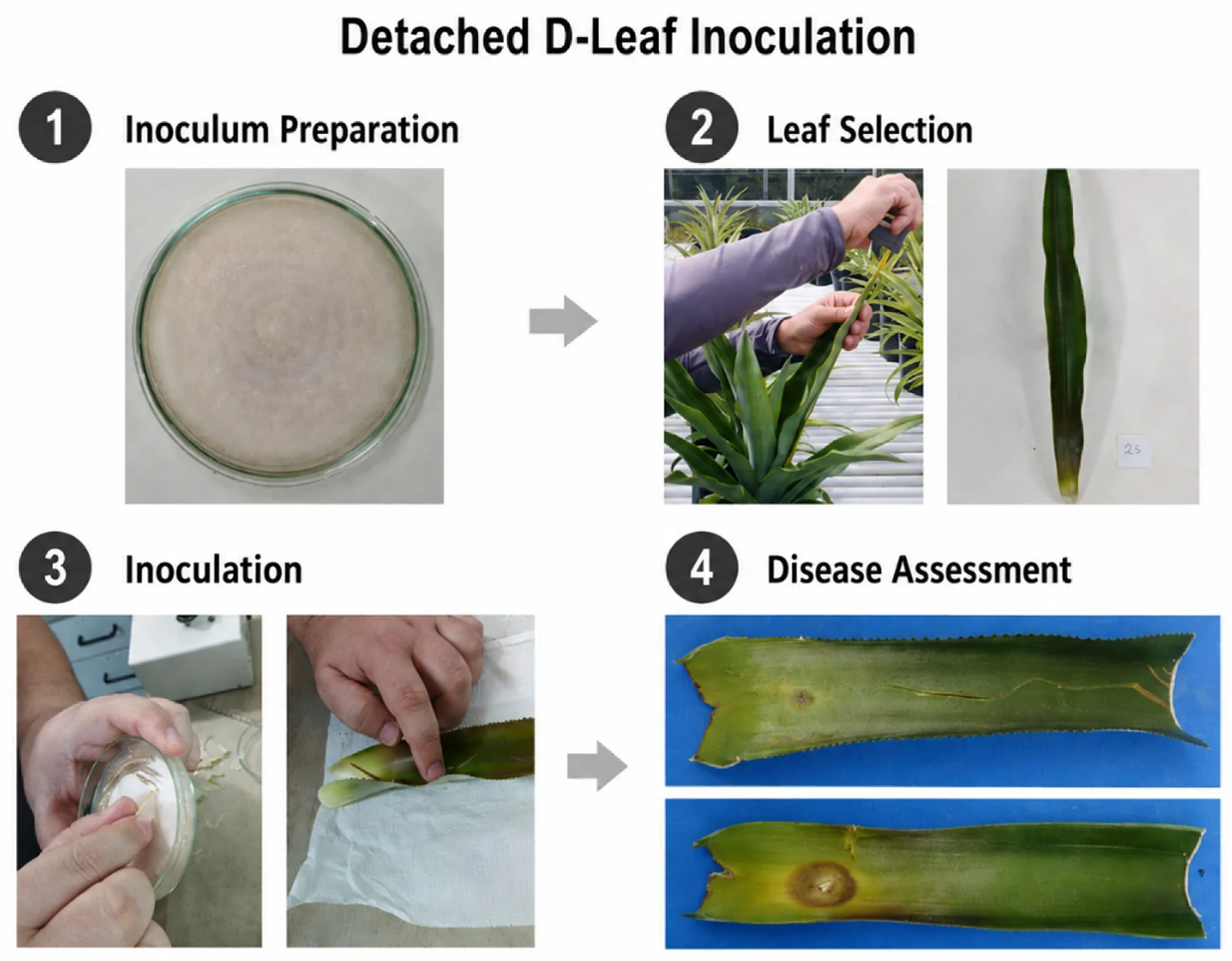
Steps of the artificial inoculation and resistance-assessment procedure using detached pineapple D leaves. (1) Culture of *F. guttiforme* isolate CF/UENF512 on potato dextrose agar; (2) collection, surface disinfestation, and standardization of the D leaf; (3) inoculation by superficial puncture at the transition between nonchlorophyllous and chlorophyllous tissues using a sterile wooden toothpick carrying mycelium from the margin of an actively growing colony; and (4) assessment of lesion development by image analysis using ImageJ software (versão 1.54k, National Institutes of Health, EUA).

**Figure 2:**
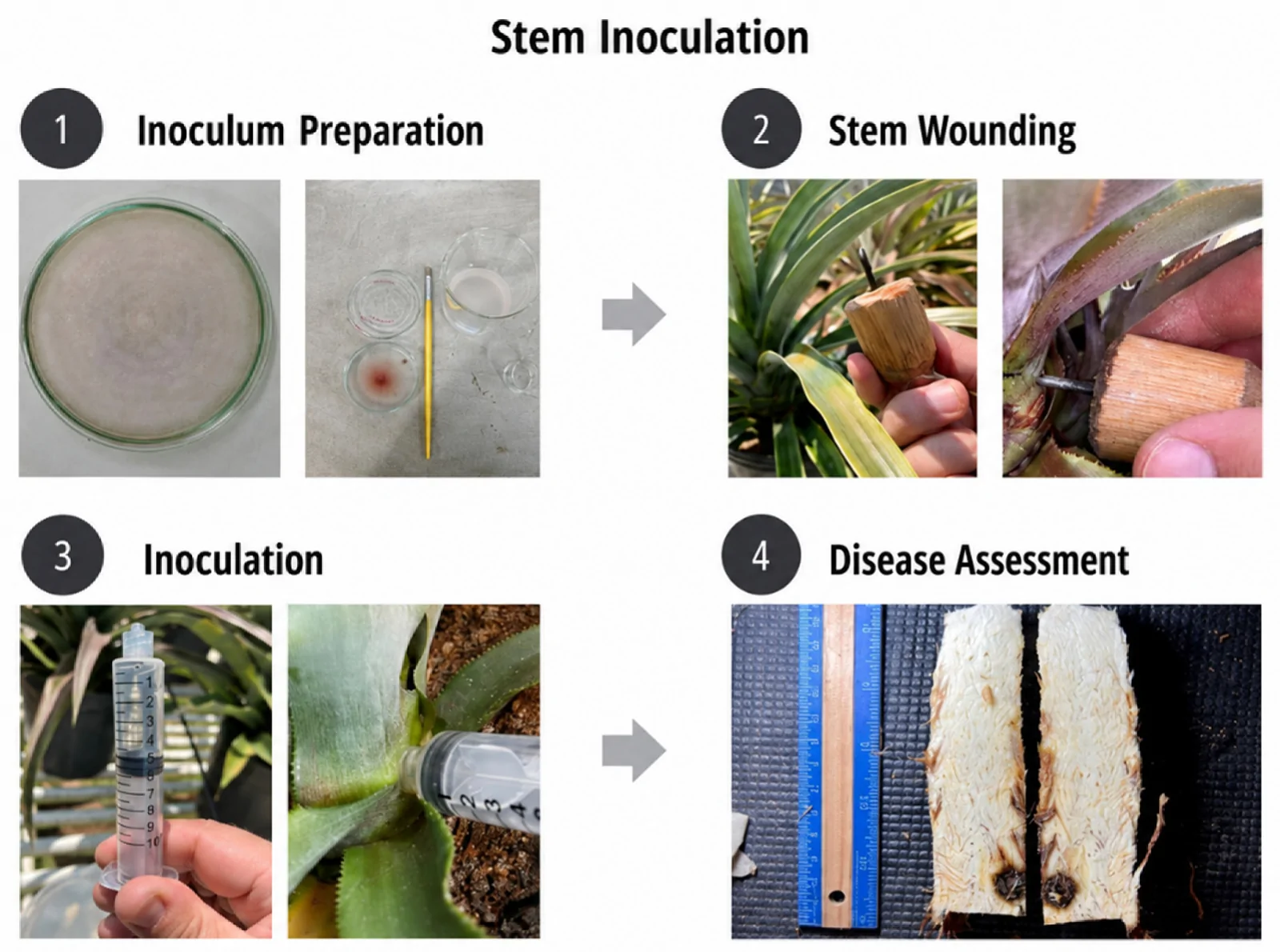
Steps of the artificial inoculation procedure for *F. guttiforme* in pineapple stems. (1) Culture of isolate CF/UENF512 on potato dextrose agar and preparation of the conidial suspension; (2) puncture of the basal stem region using a metal pin fitted with a depth limiter; (3) application of the conidial suspension to the opening using a syringe; and (4) longitudinal sectioning of the stem, exposure of the internal lesion, and image acquisition with a metric scale for measuring lesion area using ImageJ software (versão 1.54k, National Institutes of Health, Bethesda, MD, USA).

**Figure 3:**
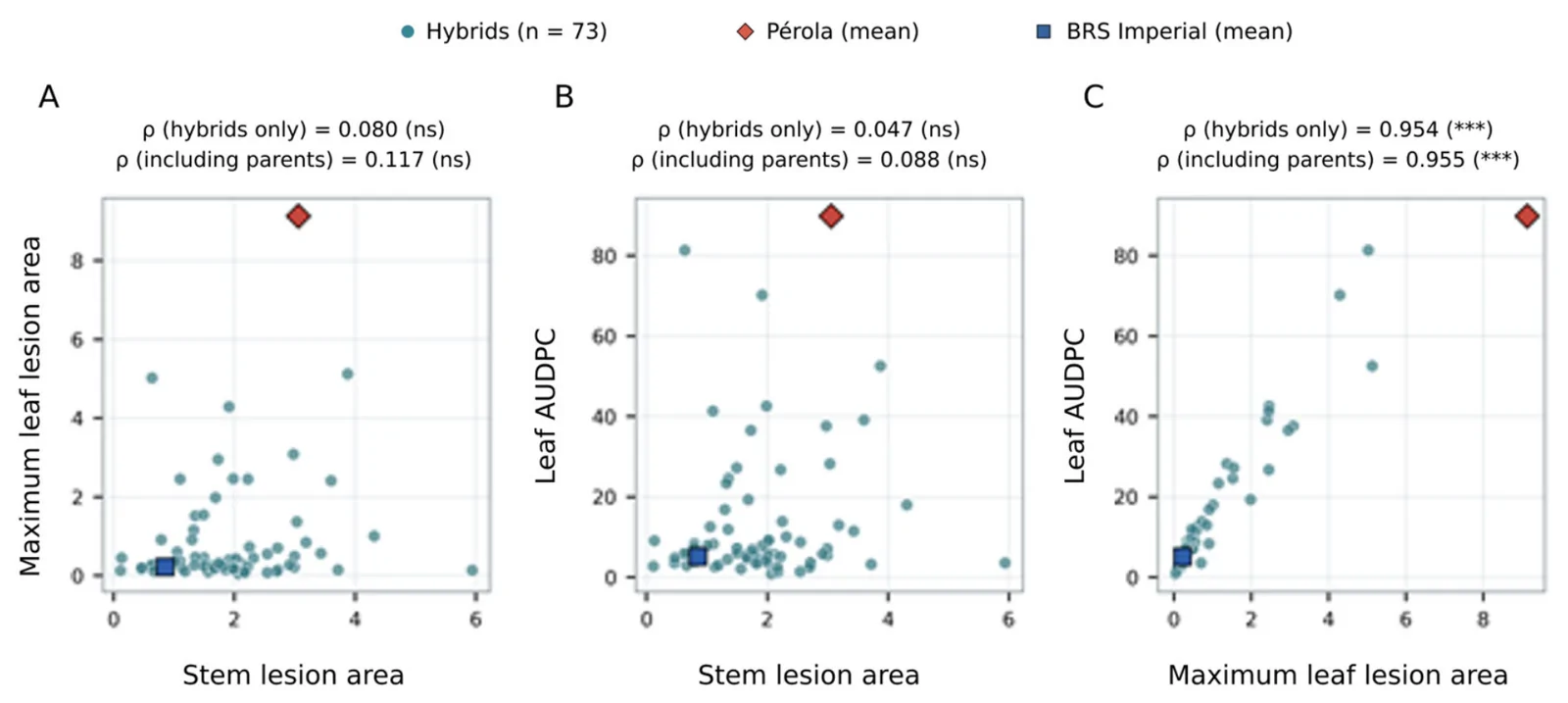
Relationships among disease-response variables in pineapple genotypes inoculated with *Fusarium guttiforme*: (**A**) stem lesion area versus maximum leaf lesion area; (**B**) stem lesion area versus the area under the disease progress curve (AUDPC) in leaves; and (**C**) maximum leaf lesion area versus leaf AUDPC. Circles represent the 73 segregating individuals; the diamond and square represent the mean values of ‘Pérola’ and ‘BRS Imperial’, respectively. The *p* values were adjusted using the Benjamini–Hochberg procedure. ***, pFDR < 0.001; ns, not significant.

**Figure 4:**
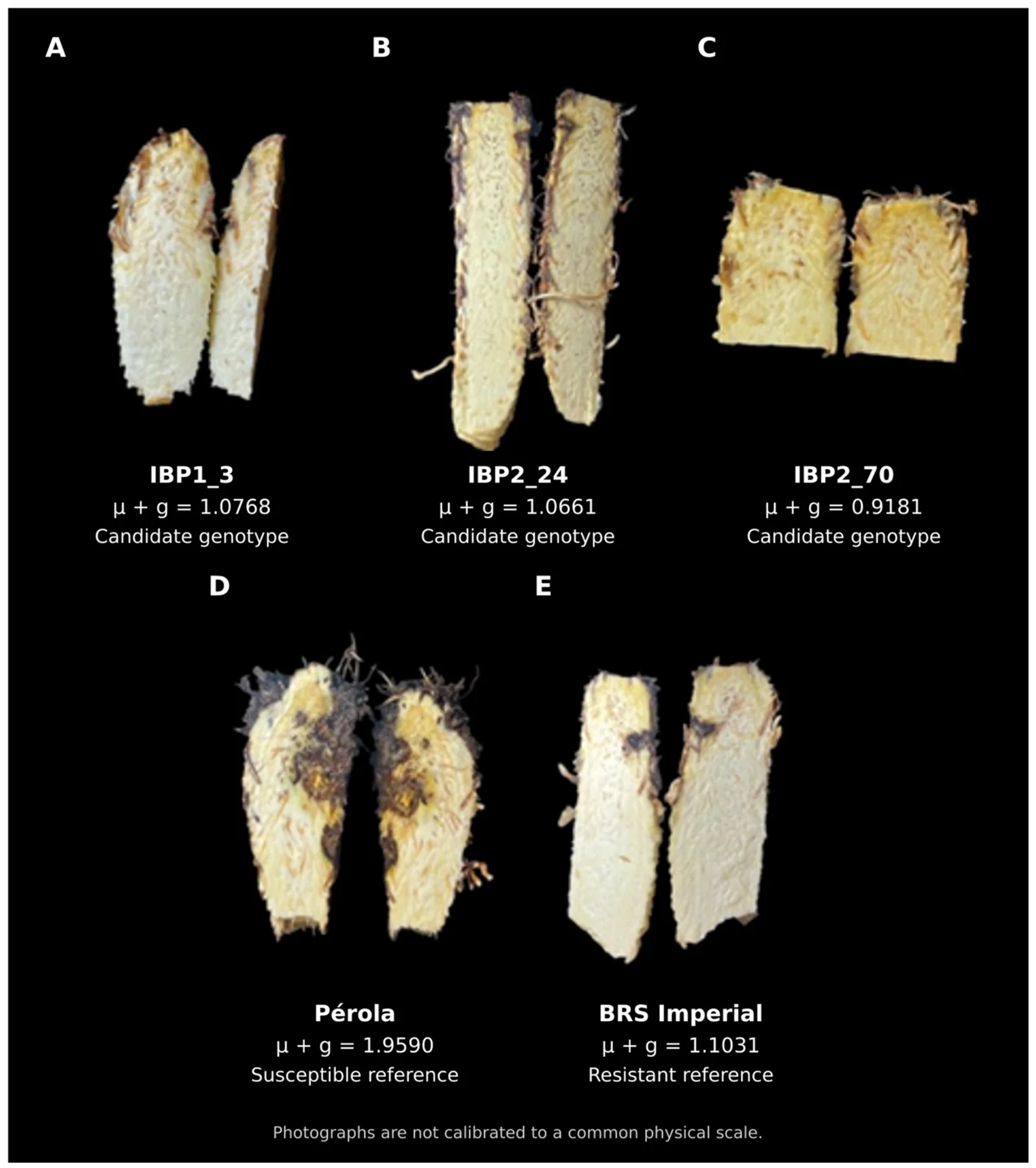
Representative internal stem lesions observed 120 days after inoculation with Fusarium guttiforme. (**A**) IBP1_3; (**B**) IBP2_24; (**C**) IBP2_70; (**D**) susceptible parental cultivar ‘Pérola’; and (**E**) resistant parental cultivar ‘BRS Imperial’. Adjusted genotypic values (μ + g) for stem lesion area are indicated for each genotype. Candidate identification was based on adjusted genotypic values obtained by REML/BLUP analysis and not solely on visual inspection of lesion appearance.

**Table 1:** Deviance analysis for traits related to resistance to *Fusarium guttiforme* in 75 pineapple genotypes, including 73 segregating individuals and the parental cultivars ‘Pérola’ and ‘BRS Imperial’.

Trait	Full-Model Deviance	Deviance Without Genotype	LRT—Genotype	Deviance Without Block	LRT—Block
Maximum leaf lesion area	234.52	244.38	9.86 **	235.11	0.59 ^ns^
Leaf AUDPC	595.80	604.25	8.45 **	595.87	0.07 ^ns^
Stem lesion area	80.35	87.65	7.30 **	81.52	1.17 ^ns^

Note: LRT corresponds to the difference between the deviance of the reduced model and that of the full model. **, significant at the 1% probability level; ^ns^, not significant.

**Table 2:** Variance components and coefficients of determination for traits related to resistance of pineapple genotypes to *Fusarium guttiforme*.

Trait	Mean	Vg	Vblock	Ve	Vf	h^2^g	c^2^block	c^2^res
Maximum leaf lesion area	0.96	2.23	0.27	4.64	7.14	0.31	0.04	0.65
Leaf AUDPC	26.75	247.20	7.97	542.24	797.41	0.31	0.01	0.68
Stem lesion area	2.16	0.69	0.05	0.29	1.03	0.67	0.05	0.28

Note: Vg, genotypic variance; Vblock, block variance; Ve, residual variance; Vf, total phenotypic variance; h^2^g, coefficient of determination of genotypic effects; c^2^block, coefficient of determination of block effects; c^2^res, proportion of residual variance.

**Table 3:** Predicted genotypic effects (g) and adjusted genotypic values (μ + g) for stem lesion area in 73 segregating individuals and the parental cultivars ‘BRS Imperial’ and ‘Pérola’ at 120 days after inoculation with *Fusarium guttiforme*.

Genotype	g	μ + g	Genotype	g	μ + g	Genotype	g	μ + g
**IBP2_70**	**−1.2468**	**0.9181**	P28I_41	−0.2715	1.8934	P36I_3	0.2037	2.3686
**IBP2_24**	**−1.0988**	**1.0661**	P30I_25	−0.2545	1.9104	IBP2_69	0.2226	2.3875
**IBP1_3**	**−1.0881**	**1.0768**	P23I_55	−0.2515	1.9134	P28I_18	0.2265	2.3914
**BRS Imperial**	**−1.0618**	**1.1031**	P28I_5	−0.2423	1.9226	P27I_8	0.2377	2.4026
P23I_17	−0.8934	1.2715	IBP2_44	−0.2323	1.9326	IBP2_31	0.2478	2.4127
IBP2_74	−0.8588	1.3061	IBP2_16	−0.2297	1.9352	IBP1_15	0.2986	2.4635
P30I_50	−0.8521	1.3128	**Pérola**	**−0.2059**	**1.9590**	P30I_4	0.3593	2.5242
IBP2_14	−0.7709	1.3940	P36I_9	−0.1989	1.9660	IBP2_61	0.3604	2.5253
P28I_7	−0.7358	1.4291	P28I_27	−0.1822	1.9827	IBP2_35	0.3756	2.5405
P28I_42	−0.7131	1.4518	P28I_24	−0.1715	1.9934	IBP2_33	0.4418	2.6067
IBP2_2	−0.6863	1.4786	P36I_5	−0.1519	2.0130	P28I_13	0.4672	2.6321
IBP2_38	−0.6259	1.5390	IBP2_4	−0.1393	2.0256	IBP2_8	0.4766	2.6415
P36I_8	−0.6234	1.5415	P36I_10	−0.1167	2.0482	IBP2_40	0.4819	2.6468
IBP2_60	−0.5653	1.5996	P27I_5	−0.0852	2.0797	P28I_26	0.6626	2.8275
IBP2_46	−0.5359	1.6290	IBP2_42	−0.0843	2.0806	P28I_2	0.6734	2.8383
P28I_35	−0.4984	1.6665	IBP2_18	−0.0323	2.1326	P28I_9	0.7132	2.8781
P30I_45	−0.4718	1.6931	P36I_16	−0.0210	2.1439	IBP2_3	0.7973	2.9622
P36I_14	−0.4105	1.7544	IBP2_75	0.0125	2.1774	P28I_17	0.8062	2.9711
IBP2_21	−0.4056	1.7593	P30I_24	0.0256	2.1905	IBP2_27	0.9139	3.0788
P30I_2	−0.3906	1.7743	IBP2_77	0.0286	2.1935	IBP2_43	1.1802	3.3451
P23I_51	−0.3818	1.7831	IBP1_4	0.0619	2.2268	IBP2_34	1.1937	3.3586
IBP1_18	−0.3750	1.7899	IBP1_20	0.0734	2.2383	IBP2_36	1.2224	3.3873
IBP2_17	−0.3732	1.7917	P36I_11	0.1005	2.2654	P30I_36	1.3849	3.5498
P27I_2	−0.2834	1.8815	P30I_3	0.1310	2.2959	IBP2_9	1.6923	3.8572
P28I_1	−0.2740	1.8909	P36I_12	0.1872	2.3521	P30I_21	2.8311	4.9960

Note: g, genotypic effect predicted by BLUP; μ + g, adjusted genotypic value calculated using μ = 2.1649. Lower values indicate smaller lesion areas and a more favorable response to inoculation. Candidates and parental cultivars are shown in bold.

## Data Availability

The data presented in this study are available on request from the corresponding author.

## Abbreviations

The following abbreviations are used in this manuscript:

AUDPC	Area under the disease progress curve
PDA	Potato dextrose agar
BLUP	Best linear unbiased prediction
CAPES	Coordenação de Aperfeiçoamento de Pessoal de Nível Superior
CNPq	Conselho Nacional de Desenvolvimento Científico e Tecnológico
FAPERJ	Fundação Carlos Chagas Filho de Amparo à Pesquisa do Estado do Rio de Janeiro
FDR	False discovery rate
LRT	Likelihood-ratio test
NPK	Nitrogen, phosphorus, and potassium
REML	Restricted maximum likelihood
UENF	Universidade Estadual do Norte Fluminense Darcy Ribeiro
